# The evolution of traditional Chinese medicine education policies in China: themes, characteristics, and experiences—a historical institutionalism analysis based on 197 policy texts

**DOI:** 10.3389/fpubh.2025.1692198

**Published:** 2026-01-05

**Authors:** Fan Yang, Tao Yang, Yong Li, Di Wang

**Affiliations:** 1 School of Management, Chengdu University of Traditional Chinese Medicine, Chengdu, Sichuan, China; 2 College of Teacher Education, East China Normal University, Shanghai, China; 3 School of Basic Medical Sciences, Chengdu University of Traditional Chinese Medicine, Chengdu, Sichuan, China; 4 College of Pharmacy, Chengdu University of Traditional Chinese Medicine, Chengdu, Sichuan, China

**Keywords:** China, historical institutionalism, policy evolution, TCM education policy, text analysis

## Abstract

**Background:**

This study examines the historical evolution of Chinese TCM education policies since 1949, analyzing the characteristics of each developmental stage, their underlying driving mechanisms, and their implications for modernizing traditional medical education.

**Methods:**

A mixed-methods approach was employed, combining quantitative content analysis (using NVivo) of 197 policy documents with qualitative analysis grounded in historical institutionalism.

**Results:**

The evolution is categorized into four distinct stages: 1949–1977, 1978–1999, 2000–2011, and 2012–present. This progression follows a trajectory from “standardization” to “distinctiveness,” then to “systematization,” and finally toward “internationalization.” Key driving mechanisms include path dependence and responses to critical junctures.

**Conclusion:**

Changes in TCM education policy arise from the interplay of national strategic needs, cultural heritage preservation, and global competition. Future reforms should prioritize strengthening clinical practice education, enhancing cultural protection within the curriculum, and fostering cross-disciplinary collaboration. This study offers an institutional paradigm for modernizing traditional knowledge systems.

## Introduction

### Global context and policy challenges

In the wave of global medical education reform, the standardization of traditional medicine education has emerged as a critical issue in transforming international health systems. A World Health Organization (WHO) report highlights that 75% of countries face significant challenges in integrating traditional and modern medical curricula, reflecting the inherent complexity of harmonizing diverse knowledge systems within contemporary educational frameworks (1).

### China’s unique position in TCM education

As a nation with a millennia-old tradition of traditional Chinese medicine (TCM), China occupies a distinctive role in global traditional medical education. It hosts the world’s largest TCM education system, comprising over 40 specialized institutions that enroll more than 300,000 students annually (2), a scale unparalleled by other traditional medical systems. However, this systemic prominence coexists with profound internal contradictions. For instance, while the Law on Traditional Chinese Medicine (2017) mandates “equal emphasis on TCM and Western medicine” (3), clinical curricula in TCM institutions consistently allocate over 50% of teaching hours to Western medical content (2), highlighting a persistent tension between policy rhetoric and educational practice.

### Research gaps and theoretical framework

Existing studies on TCM education policies have primarily focused on post-1978 reforms using descriptive or comparative approaches. For example, Wang et al. (4) conducted a quantitative analysis of 43 national policies from 1997 to 2021, identifying trends in curriculum standardization but offering limited theoretical explanation for the underlying policy shifts. Similarly, WHO comparisons of accreditation systems between China and India have often overlooked the historical institutional factors that shaped China’s unique policy trajectory. At the micro-level, studies such as that by Zhou et al. (5) have examined postgraduate education policies but failed to adequately contextualize these institutional changes within broader political-economic transformations.

Three critical gaps persist in the current literature. First, there is a issue of temporal discontinuity, as over 80% of existing research commences its analysis after 1978, thereby neglecting the foundational policies from the 1950s that established TCM education as a state-sanctified system. Second, methodological limitations are evident; while policy text analysis is common, it remains predominantly descriptive, with no studies applying computational tools like NVivo for thematic modeling and latent pattern discovery. Third, a theoretical disconnect exists. Unlike the broader field of higher education studies, which frequently employs historical institutionalism, TCM policy research lacks robust theoretical frameworks to link macro-institutional structures to specific policy outcomes.

### Research objectives

Against this backdrop, this study employs historical institutionalism to analyze 197 national TCM education policy texts from 1949 to 2023, aiming to bridge the identified research gaps. Guided by its core questions, this research seeks to: (1) trace the evolutionary pathway of TCM education policies by identifying key transformations across distinct historical stages; (2) uncover the institutional drivers-including both macro-structures and micro-level dynamics-that have shaped major policy changes; and (3) characterize the stage-specific policy priorities and assess their alignment with broader societal needs.

By systematically mapping policy trajectories and their contextual determinants, this research contributes theoretical insights to the literature on institutional adaptation within traditional knowledge systems. Furthermore, it provides practical guidance for policymakers navigating the complex tension between tradition and modernity in medical education, offering a critical framework for optimizing TCM education policy design in an era of globalization and evidence-based healthcare.

## Materials and methods

### Data sources

The selection of policy texts was guided by three stringent criteria to ensure the dataset’s authority, comprehensiveness, and relevance. First, the principle of authority mandated that all documents be issued exclusively by national or ministerial-level bodies, thereby excluding provincial-level policies to maintain a consistent, macro-level perspective. Second, for comprehensiveness, a wide spectrum of policy types was incorporated, including policy plans, laws and regulations, administrative decrees, implementation guidelines, development outlines, official notices, and statistical reports. This diverse collection was essential to capture the full evolution of TCM education policies across different historical periods. Finally, the criterion of relevance focused on including TCM-specific education policies while also incorporating overarching national medical education policies that had direct implications for TCM.

The systematic search for these documents was conducted across four primary categories of sources.

1. The process began with a review of official compilations of TCM education policies, such as Selected Higher Education Policies for Traditional Chinese Medicine in China (6).
2. Subsequently, major legal and academic databases, including PKULAW and CNKI, were exhaustively queried. In PKULAW, a targeted search was performed within the “Central Regulations” module. Key search terms included “Chinese medicine,” “traditional Chinese medicine,” “education,” “talent cultivation” and “institutions.” These terms were combined using Boolean operators and searched within the titles, abstracts, and full texts of policy documents. The search was limited to documents issued between 1949 and 2023 by key authorities such as the Ministry of Education, the National Health Commission (and its predecessors), and the National Administration of Traditional Chinese Medicine. The results were screened by title and abstract, and full texts of all relevant documents (laws, regulations, departmental rules, and normative documents) were retrieved. In CNKI, the search focused on identifying scholarly literature to inform the analytical framework and contextual background. Searches were conducted in the core journals collection using subject terms such as “TCM education policy,” “development of TCM education” and “education in the TCM Law.” Publications were filtered by CSSCI and core journal status to ensure academic quality. Seminal works were identified through citation tracking.
3. Official government portals, specifically those of the Ministry of Education and the National Administration of Traditional Chinese Medicine (NATCM), were also consulted to retrieve primary policy documents.
4. Finally, supplementary sources were reviewed, which involved tracking indexed references from key academic journals in the field to ensure no critical policies were overlooked.

To contextualize policy changes, this study also drew on: theoretical foundations: classic works on education policy (7) and historical institutionalism (8), recent research on TCM curriculum evolution (9), these academic literatures provide this paper with rich theoretical support and research perspectives. At the same time, this paper also makes use of relevant archival materials and historical documents, especially those related to the historical development of traditional Chinese medicine education. These materials are helpful for restoring the historical background and gaining an in-depth understanding of the specific contexts in which the policies were formulated and implemented.

### Research methods and tools

#### Research method: historical institutionalism

This study employs historical institutionalism as its core research method. This approach places significant emphasis on the crucial roles played by historical contexts and institutional arrangements in the process of policy evolution (10). Based on the analytical framework of historical institutionalism and combined with the development history of traditional Chinese medicine (TCM) education policies in China, we divide this development process into different stages and conduct a comprehensive analysis from aspects such as the background of the times, policy characteristics, and critical turning points. Firstly, in terms of institutional origins, we trace back to the establishment of the first batch of TCM education institutions in the early days of the People’s Republic of China in the 1950s. By analyzing the early policies, we aim to understand the political, economic, and social factors that influenced the birth of the TCM education system. These early institutional settings laid the foundation for the subsequent development of TCM education policies.

Secondly, we focus on the path dependence of policy development. By tracing the changes in policies over time, we identify how previous policies have influenced subsequent ones. For example, the early curriculum setting policies in TCM education had a lasting impact on the curriculum structure in the following decades. Under the influence of the country’s overall development strategy and international medical trends, the initial emphasis on traditional TCM theories gradually evolved to incorporate modern medical knowledge.

In addition, historical institutionalism requires the identification of critical nodes in policy evolution. Events such as the reform and opening-up in 1978 and the promulgation of the Traditional Chinese Medicine Law are regarded as critical turning points. At these moments, external shocks or internal policy adjustments led to significant changes in TCM education policies. By analyzing the policy-making processes at these critical nodes, the interactions among stakeholders, and the resulting policy outcomes, we can gain a better understanding of the mechanisms driving the evolution of TCM education policies.

#### Research tool: NVivo qualitative data analysis

To conduct an in-depth analysis of the themes and priorities in TCM education policies across different stages, this study employed NVivo, a robust software package designed for qualitative and mixed-methods research. The use of NVivo enabled a systematic and replicable analytical process that combined quantitative text mining with qualitative thematic exploration. The methodology comprised four key steps that leveraged specific functionalities of the software.

First, the stage of Document Management and Import involved systematically uploading a total of 197 policy texts into the NVivo project to create a centralized and organized database. All texts were converted into standardized machine-readable formats (PDF or Word) to ensure consistency for subsequent analysis.

Subsequently, Thematic Coding and Framework Analysis was conducted. Guided by the principles of historical institutionalism, a hierarchical coding scheme was developed within NVivo. The qualitative content analysis followed a structured coding process. Two researchers independently performed close-reading coding on the same subset of policy documents (approximately 20% of the total), categorizing text segments into a predefined codebook containing themes such as historical context, policy characteristics, and critical junctures. To ensure reliability, this process involved double coding. Any discrepancies in code application were discussed and resolved through consensus between the two coders. After achieving a satisfactory inter-coder reliability, the remaining documents were divided and coded by a single researcher, with ongoing consultations held for ambiguous segments. This rigorous process facilitated a structured and reliable qualitative analysis of the policy content.

The third step, Quantitative Text Mining via Word Frequency Analysis, served to complement the qualitative coding. In this phase, NVivo’s built-in word frequency query tool was utilized to quantify the occurrence of key terms across the entire corpus and within different policy eras. The resulting temporal comparisons provided objective, quantitative evidence for identifying shifts in policy emphasis.

Finally, the process moved to Latent Theme Discovery through Automated Theme Modeling. This step utilized NVivo’s “Themes” automation feature, which performs a co-occurrence analysis of codes and keywords to identify latent, underlying topic clusters. This advanced analysis helped uncover non-obvious patterns and connections within the data that might not have been apparent through manual coding alone.

By integrating historical institutionalism’s stage-based framework with NVivo’s multi-faceted analytical capabilities, this study systematically uncovered evolutionary patterns in TCM education policies. The triangulation of insights from distinct methods-manual qualitative coding, quantitative frequency counts, and automated latent theme extraction-provided a robust and multi-dimensional evidence base. This integrated approach within NVivo effectively demonstrates how institutional legacies, political shifts, and societal demands have collectively shaped TCM education policy trajectories over seven decades.

## Results

**Figure 1:**
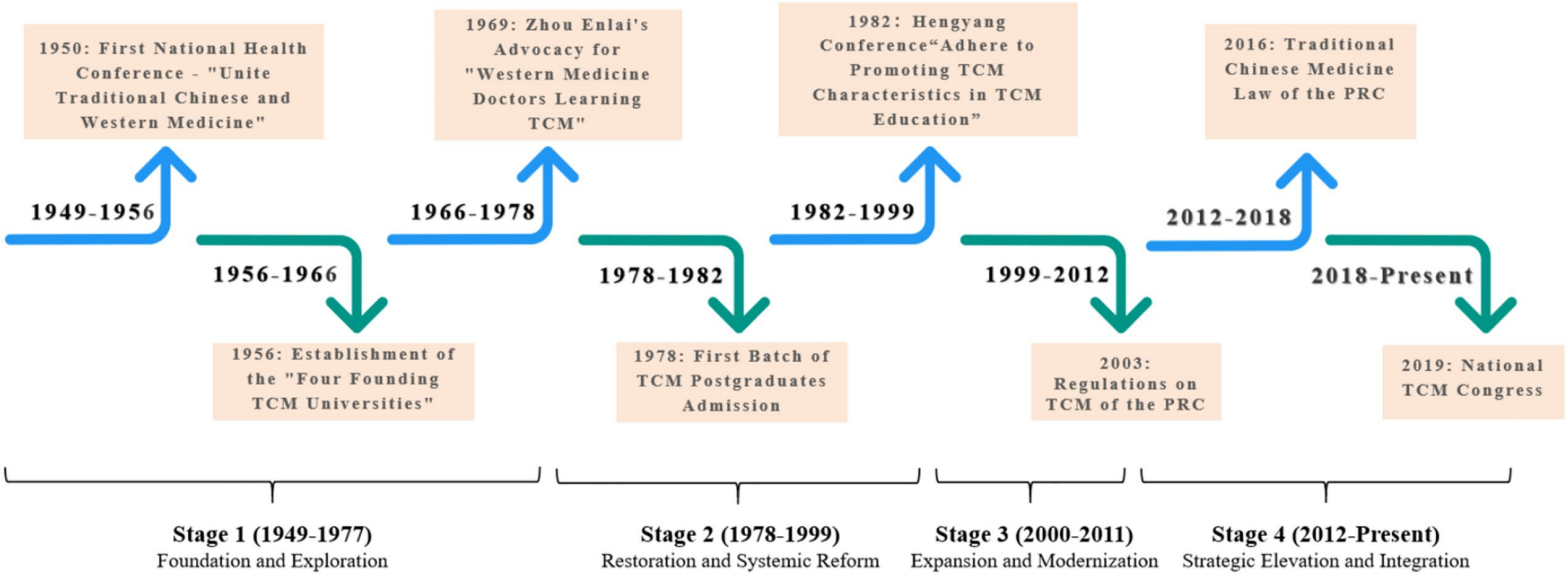
Historical timeline of TCM education policy evolution in China (1949–present).

This study has collected a comprehensive dataset consisting of 197 policy texts. These texts span from the early days of the People’s Republic of China in the 1950s to the present, providing a long-term perspective for the study of the evolution of traditional Chinese medicine (TCM) education policies. According to the theory of historical institutionalism, institutional changes can be triggered by alterations at critical junctures. A critical juncture refers to “an important turning point in historical development that can influence the historical process, and is also known as an institutional breakpoint, a point of conflict outbreak, an inflection point, a watershed event, or a critical choice point, etc.” (11). Marked by critical junctures, dividing the development stages of China’s TCM education policies not only allows us to trace back to the driving forces and key elements of institutional changes, but also enables us to explore the impacts and trends of these changes in the future, which is conducive to revealing the important laws of institutional changes (12). Based on this, we identify the establishment of the People’s Republic of China in 1949, the convening of the Third Plenary Session of the 11th Central Committee of the Communist Party of China in 1978, the entry into the “New Century” in 2000, and the convening of the 18th National Congress of the Communist Party of China in 2012 as critical junctures. Accordingly, the development process of China’s TCM education policies is divided into the following four stages. Each stage is characterized by distinct policy themes and emblematic documents, as summarized in Figure 1, Table 1.

**Table 1:** Critical junctures and characteristic policies in TCM education evolution.

Time period	Critical juncture	Policy focus and characteristics	Number of texts	Representative policies (issuing authority, date of issuance)
1949–1977	Founding of the PRC (1949)	Founding the system, shaping the model	13	Guidelines for TCM curriculum and textbooks (MOH, 1959); Circular on educational reform in medical colleges (MOH, 1965)
1978–1999	Reform and opening-up (1978)	Restoring order, building the system	98	Opinion on teaching issues (MOH, 1978); Circular on national TCM conference (SATCM, 1982); Opinion on medical education reform (MOH, 1985) Circular on 7-year TCM program (SEC, 1991); Opinion on TCM education reform (SEC and SATCM, 1997)
2000–2011	Entering the new century (2000)	Scaling up, elevating quality	45	Opinion on TCM education (SATCM and MOE, 2003); TCM regulation (State Council, 2003); Opinion on medical education quality (MOE and MOH, 2009)
2012–present	18th CPC National Congress (2012)	Strategic support, integrated development	41	Opinion on excellence in doctor education (MOE and MOH, 2012); Guideline on TCM apprenticeship (SATCM, 2018); Guideline on medical education innovation (State Council, 2020); Implementation opinion on TCM education reform (MOE, NHC and SATCM, 2020)

The evolutionary trajectory of TCM education policy, as delineated by our analysis, unfolds across four distinct stages, each marked by a unique policy orientation. The initial stage (1949–1977) was characterized by Foundation and Exploration, focusing on establishing a modern TCM education system within the new state structure and transitioning from traditional apprenticeship to institutionalized education. This foundational period saw the introduction of inaugural regulations for TCM colleges and the formal integration of TCM into the national health plan.

Following the critical juncture of Reform and Opening-up, the second stage (1978–1999) transitioned into a phase of Restoration and Systemic Reform. Policy emphasis shifted towards restoring institutional order and standardizing TCM higher education, with concerted efforts directed at curriculum standardization, the formal establishment of academic degrees, and the systemic improvement of educational quality.

The dawn of the new century heralded the third stage (2000–2011), defined by Expansion and Modernization. Influenced by national priorities for modernization and global integration, policies during this era actively promoted the scaling up of TCM education, the enhancement of talent quality, and the fostering of innovation within the field.

The current and ongoing stage (2012–present) has entered an era of Strategic Elevation and Integration, propelled by a top-level national strategy to revitalize TCM. Consequently, recent policies have focused on building world-class TCM disciplines, deepening the integration of TCM into the broader healthcare system, and promoting the innovative development of TCM education.

The visual timeline in Figure 1 succinctly captures the four evolutionary stages, their defining critical junctures, and shifting policy priorities. This macro-level perspective is further substantiated by the detailed characteristic policies presented in Table 1.

### Themes of each stage of policy change

Guided by the framework of historical institutionalism and utilizing NVivo’s keyword extraction capabilities, this study identifies the dominant thematic evolution across the four stages of TCM education policy. The analysis reveals a clear developmental trajectory, with each stage embodying a distinct central theme reflective of its historical context.

#### Stage 1 (1949–1977): institutional foundation and exploratory models

The first stage (1949–1977) was characterized by the theme of Foundation and Exploration. Following the founding of the People’s Republic of China, the primary policy focus was on establishing a modern, state-sanctioned TCM education system, transitioning it from traditional apprenticeship models to formal institutionalized education. The word frequency analysis of policy texts from this period, as detailed in Table 2, highlights keywords pertinent to this foundational phase.

**Table 2:** Keywords and their word frequencies (top 10) in traditional Chinese medicine education policies in China from 1949 to 1977.

Keyword	Word frequency
Student	59
Teaching	45
Work	38
Learning	33
Education	33
Thought	30
Class hour	24
Medicine	23
Health	21
Traditional Chinese medicine (in a more general sense)	20

This stage was characterized by three core features: the reconstruction and standardization of educational frameworks, the integration of traditional knowledge with innovative practices, and periods of setback alongside remarkable perseverance from the TCM community. During this period, the overarching goal was to integrate TCM into the modern education system while enhancing its scientific rigor and standardization. Policy initiatives focused on advancing the professional competence of TCM practitioners, primarily through the establishment of specialized TCM colleges and advanced training programs, the promotion of integrated traditional Chinese and Western medicine, and the acceleration of TCM modernization efforts. Furthermore, greater emphasis was placed on practical skills development, particularly through the traditional apprentice-mentoring system and hands-on clinical practice that reinforced theoretical learning. Although TCM education suffered severe disruptions during the Cultural Revolution, the continuity of core national policies and the unwavering resilience of the TCM community safeguarded the fundamental pillars of TCM education from complete collapse. This persistence not only preserved critical educational traditions but also accumulated valuable experience that would provide robust support for the subsequent reconstruction and sustainable development of the sector.

Higher TCM education took shape in the early years of the People’s Republic of China, a period defined by specific historical contexts that gave rise to several critical imperatives. Politically, the newly established nation sought to break away from the outdated educational framework of the previous regime and build a system aligned with socialist values. In the public health and epidemic prevention sectors, there was an urgent need to improve the scientific literacy of TCM practitioners to address national health challenges effectively. Additionally, in the implementation of the Party’s TCM policies, it became crucial to rectify misalignments and inconsistencies in policy execution to ensure effective governance. Consequently, the creation of a new, standardized TCM education system became unavoidable (13). To drive socialist transformation and national modernization, the central government established a planned economic system centered on public ownership, pooling national resources to support large-scale economic construction and comprehensive social reforms. Education was recognized as a vital component of socialist development, and TCM education received significant strategic attention within this framework. The institutional environment of this era-characterized by coordinated policy support and systematic institutional guarantees-created favorable conditions for the restoration, restructuring, and sustained advancement of TCM education in China.

#### Stage 2 (1978–1999): restoring order and systemic standardization

This period is defined as the phase of restoring operational order and implementing systemic standardization for China’s TCM education. Word frequency analysis results for key terms in China’s TCM education policy documents during this stage are presented in Table 3.

**Table 3:** Keywords and their word frequencies (top 10) in traditional Chinese medicine education policies in China from 1978 to 1999.

Keyword	Word frequency
Education	2,347
Traditional Chinese medicine (in a more general sense)	1808
Teaching	1,186
Work	1,135
Traditional Chinese medicine	1,054
Specialty	1,027
Development	962
School	846
Medicine	787
Cultivation	676

In 1978, following the full-scale launch of China’s reform and opening-up policy, the country entered a new phase of rapid economic and social development. In the policy domain, the Chinese government encouraged diverse forms of international opening-up and reforms to the economic system, thereby driving comprehensive innovation and development across all sectors of society. Within this institutional context, the education sector also underwent substantial transformations: the government introduced a series of education reform policies focused on enhancing educational quality and advancing educational modernization. In 1985, the Decision of the Central Committee of the Communist Party of China on the Reform of the Educational System outlined the overarching framework for deepening education reform. Building on this foundation, the 1993 Outline for China’s Educational Reform and Development further refined and clarified the direction of education system reform. The overall institutional environment of this period was shaped by the dual backdrops of reform and opening-up and the national modernization drive. Policies during this time emphasized the integration of economic development and social progress, with education reform and economic system reform mutually reinforcing one another. Against this backdrop, TCM education underwent restoration, development, and enhancement, gradually completing a transformation from a traditional model to a modernized one, and from a domestically focused approach to an internationally oriented one.

During this stage, the government released a series of guiding documents and policy initiatives that clearly defined the core tasks, training objectives, program offerings, and curriculum content for TCM education. These policies also encouraged the integration of traditional Chinese and Western medicine, strengthened clinical teaching practices, and advanced the reform and development of higher education institutions specializing in TCM. The primary focus of these policies was to reconstruct and further develop the TCM education system—with a strong emphasis on preserving the unique characteristics of TCM while advancing the integration of traditional Chinese and Western medicine. Governance of the TCM education sector was strengthened through the establishment of specialized regulatory bodies, such as the State Administration of Traditional Chinese Medicine, which in turn facilitated the systematization, standardization, and modernization of TCM education. The evolving nature of these policy adjustments reflects the developmental trajectory of TCM education since the onset of reform and opening-up. This trajectory evolved through three key phases: first, establishing and refining educational institutions and systems; second, constructing a TCM education system with distinct disciplinary features; and third, deepening reforms in TCM education and teaching practices. This progression demonstrates a developmental trend that gradually deepened from external institutional building to internal quality enhancement, and from targeted improvements in specific areas to comprehensive systemic advancement (14).

#### Stage 3 (2000–2011): quantitative expansion and modernization transition

This period is designated as the stage of quantitative expansion and systematized transition toward modernization for China’s TCM education. Word frequency analysis results for key terms in China’s TCM education policy documents during this stage are presented in Table 4.

**Table 4:** Keywords and their word frequencies (top 10) in traditional Chinese medicine education policies in China from 2000 to 2011.

Keyword	Word frequency
Education	2,536
Traditional Chinese medicine	2,435
Continuing	968
Development	865
Management	779
Specialty	710
Work	629
Traditional Chinese medicine (in a more general sense)	628
Medicine	627
Cultivation	615

At the turn of the new century, China’s economy continued its rapid growth, with incomes of both urban and rural residents on a steady rise and the overall living standards of society improving significantly. Such sustained economic growth provided a solid material foundation for the advancement of various social undertakings. During this time, the government placed greater emphasis on advancing the implementation of the Scientific Outlook on Development, highlighting the people-oriented development philosophy and promoting the all-round progress of the economy, politics, culture, and society. In the field of education, the state increased its investment in educational initiatives and introduced a series of education reform measures, with the goals of enhancing educational quality, promoting educational equity, and advancing the internationalization of education. In the healthcare sector, the State Council issued the Opinions on Deepening the Reform of the Medical and Health Care System in 2009, launching a new round of national medical reform (hereafter referred to as the “New Medical Reform”). This reform framework put forward new requirements for the development of TCM while also creating new opportunities for its advancement. During this stage, China’s rapid economic and social development, the deepening of reforms in the education and healthcare systems, and the introduction of a series of TCM-specific policies collectively formed the institutional environment underpinning the development of TCM education.

Throughout this period, the state clarified the reform direction and specific measures for TCM education through the release of a series of policy documents. The National Administration of Traditional Chinese Medicine (NATCM) and the Ministry of Education (MOE) continuously refined the systems for postgraduate education and continuing education in TCM, optimized the hierarchical structure of TCM-related medical education, and accelerated the cultivation of high-level TCM professionals. The core focuses of these policies included enhancing the quality of TCM education, promoting the internationalization of TCM education, strengthening talent development, and refining the TCM education system. The characteristics of policy adjustments during this period were primarily manifested in several key dimensions, including the modernization of TCM education, its internationalization, institutional reform within the sector, and targeted policy support for TCM development.

#### Stage 4 (2012–present): strategic elevation and integrated innovation

This period is defined as the stage of strategic elevation and integrated innovation for China’s TCM education, with a core focus on standardization. Word frequency analysis results for key terms in China’s TCM education policy documents during this stage are presented in Table 5.

**Table 5:** Keywords and their word frequencies (top 10) in traditional Chinese medicine education policies in China from 2012 to the present.

Keyword	Word frequency
Traditional Chinese medicine	2,296
Traditional Chinese medicine (in a more general sense)	2,258
Education	1,657
Cultivation	1,401
Training	1,357
Talent	1,305
Medicine	1,200
Clinical	1,174
Specialty	971
Development	890

Since 2012, China has entered a new era of national development. During this period, the government has continued to advance economic transformation, industrial upgrading, and social progress-while placing prominent emphasis on the Healthy China Initiative and the goal of building a moderately prosperous society in all respects. The state has also attached even greater importance to TCM undertakings, releasing a series of policy documents focused on inheriting and innovating TCM, as well as advancing its modernization, standardization, and internationalization. In the field of education, the state has rolled out policies to advance the “Double First-Class” initiative (a national strategy to develop world-class universities and first-class disciplines), driving the modernization of higher education and further enhancing educational quality. These higher education policies have offered robust support for the development of TCM institutions. Meanwhile, the Ministry of Education (MOE) and the National Administration of Traditional Chinese Medicine (NATCM) have strengthened their joint cooperation to advance the refinement and enhancement of the TCM education system. Through the integration of medical and educational resources, the two authorities have deepened TCM education reform, with the aim of cultivating high-caliber TCM professionals. The institutional environment during this period is centered on the Healthy China Initiative. It emphasizes the inheritance, innovation, and high-quality development of TCM in the new context, seeks to further promote the integration of TCM education with modern technologies and international standards, and works to comprehensively elevate the level and global influence of TCM education.

From 2012 to the present, the evolving characteristics of TCM education policies have been primarily manifested in four key dimensions: institutionalization, modernization, the integration of medical and educational resources, and high-quality development. By issuing a series of laws, regulations, and policy documents, the state has accelerated the legalization of TCM education, formulated national standards and quality criteria for TCM education, and further advanced its standardization. In terms of modernization, policies focus on innovating the TCM talent-cultivation model, emphasizing scientific and systematic methodologies, and enhancing TCM-related clinical and public health practice capabilities. The integration of medical and educational resources-regarded as a core strategy-seeks to comprehensively deepen TCM education reform, gradually establish and refine the TCM apprenticeship education system, and facilitate the integration of TCM inheritance and innovation.

High-quality development serves as the core goal of these policies. By strengthening the development of TCM disciplines and specialties, optimizing the talent-cultivation structure, and advancing reforms in long-term TCM education systems and vocational TCM education, the quality of TCM education has been steadily enhanced. Additionally, the state actively promotes the alignment of TCM education with international standards to further expand its global influence.

## Discussion

### Institutional origins and path dependence: the early institutional imprinting of TCM education

Historical institutionalism posits that “initial conditions” in the origin of an institution exert a profound and enduring influence on its subsequent development trajectory (15). In the early years of the People’s Republic of China, to advance the modernization of the national healthcare system, the central government moved to integrate traditional TCM apprenticeship education into a modern institutional framework. This integration was facilitated through targeted policy instruments, such as the Regulations on Organizing Advanced TCM Training Schools and Classes-a measure that laid the groundwork for formalizing TCM education (13). Two core principles established during this period integrating “traditional Chinese and Western medicine” and “scientizing TCM” formed the foundational basis for path dependence in subsequent TCM education policies. Notably, even as the 1962 Notice on Several Issues of Teaching Work in TCM Colleges emphasized the need to preserve “TCM characteristics” basic Western medicine courses remained a mandatory component of the curriculum. This seemingly contradictory arrangement reflected the early institutional pursuit of “scientific legitimacy” for TCM, a goal that persisted across policy iterations (14). This path dependence continued to manifest in later stages: for example, the 1988 Strategic Plan for TCM Education Development still prioritized “integrating traditional Chinese and Western medicine” as a key objective, providing empirical support for the “institutional stickiness” concept in historical institutionalism.

Early institutionalization of TCM education was not only reflected in the formulation of policy texts but also solidified through concrete organizational mechanisms. A pivotal example is the formal establishment of four national-level TCM colleges in 1956—located in Beijing, Shanghai, Guangzhou, and Chengdu—which marked a critical transition from fragmented, informal TCM training to systematic, standardized higher education. This process of “organizationalization” aligns with March and Olsen’s (16) theoretical framework, which defines institutions as “rules and procedures” that structure social and organizational behavior. In this case, specialized TCM colleges served as intermediaries that translated broad, vague policy goals (e.g., “modernizing TCM education”) into tangible operational frameworks, such as standardized curricula and faculty training systems (17). Furthermore, the policy focus on “curriculum standardization” during this early period—exemplified by the 1958 Opinions on Compiling Teaching Outlines and Textbooks for TCM Courses—reflected the institutional designers’ pursuit of technical rationality. This emphasis on standardization was not merely a procedural detail; it exerted a profound and long-lasting influence on the subsequent construction of TCM education evaluation systems, shaping how educational quality would be measured and monitored in later decades (18).

### Institutional breakthroughs at critical junctures: historical drivers of policy shifts

Critical juncture theory posits that major historical events or systemic changes have the capacity to disrupt established institutional paths and reshape the long-term developmental trajectories of institutions (19). This study identifies two pivotal turning points that have driven significant shifts in TCM education policy: the launch of China’s Reform and Opening-Up policy in 1978 and the initiation of the New Healthcare Reform in 2009. During the early phase of Reform and Opening-Up, the Opinions on Strengthening Higher TCM Education formally introduced the concept of a “systematic higher TCM education framework.” This policy innovation marked a critical transformation in TCM education—shifting it from a primarily vocational training model (focused on skill transmission) to a standardized degree-based education system (emphasizing theoretical depth and academic rigor). This shift aligns with Xiao’s (12) theoretical perspective, which posits that critical junctures act as catalysts for institutional reconstruction by creating windows of opportunity to break from outdated models. The 2009 Opinions on Deepening Healthcare System Reform integrated TCM into the national basic healthcare framework, redirecting policy focus from quantitative expansion to qualitative improvement, reflecting how external shocks can realign institutional trajectories.

Institutional breakthroughs at critical junctures involve more than policy text adjustments; they entail fundamental shifts in power structures and resource allocation. For example, the 2017 Traditional Chinese Medicine Law of the People’s Republic of China legally codified TCM education for the first time, marking a transition from “administrative guidance” to “legal guarantees.” This aligns with Bates’ (20) “critical juncture” theory, where institutional restructuring often occurs during windows of power imbalance or external crises. Additionally, the 2015 Strategic Plan for TCM Development (2016–2030) prioritized internationalization, reflecting globalization’s influence on policy direction (National Administration of TCM, 2016). This outward-oriented shift mirrors Wang’s (21) policy learning mechanism theory, demonstrating China’s strategy to modernize domestic institutions by incorporating global standards.

### Interest group dynamics and policy choices: microfoundations of institutional change

Historical institutionalism emphasizes the interactive logic among multiple actors within institutional fields, highlighting how the negotiations, conflicts, and collaborations between different stakeholders shape institutional development and policy outcomes (22). In the context of TCM education policy-making, the interagency interaction dynamics between the National Administration of Traditional Chinese Medicine (NATCM) and the Ministry of Education (MOE)—two core authorities with distinct mandates—have been particularly influential in guiding policy direction. For example, the 2012 Guidelines on Integrating Medical and Educational Synergy to Deepen TCM Education Reform emerged from addressing the “overemphasis on research over clinical practice” in TCM institutions while simultaneously aligning with the MOE’s “Double First-Class” initiative performance metrics. This tension became even more evident during the standardization phase: negotiations among academic associations, healthcare institutions, and government agencies during the formulation of the National Standards for TCM Specialty Education Quality resulted in a hybrid model balancing “TCM thinking cultivation” with “modern medical standards”.

Interest group interactions extend beyond government agencies to include academic communities and industry stakeholders. The 2015 Strategic Plan for TCM Development (2016–2030) explicitly incorporated “industry-university-research collaboration,” reflecting pharmaceutical enterprises’ influence on talent cultivation standards (NATCM, 2016). This cross-sectoral alignment mirrors Moe’s (23) theory of “institutions as political instruments,” where policymakers balance competing demands through rule restructuring. Similarly, the 2017 TCM Law legislative process saw negotiations between grassroots TCM practitioners and state agencies over apprenticeship certification, culminating in a dual-track system combining degree education with apprenticeship assessment (State Council, 2016). This outcome aligns with Kingdon’s (24) multiple streams framework, demonstrating how policy windows open at the confluence of problem, policy, and political streams.

### Ideational shifts and institutional reconstruction: generational transformations in policy paradigms

Institutional change involves not merely rule adjustments but also the iterative evolution of dominant ideas (25). This study reveals that TCM education policies have undergone ideational shifts from “scientization” to “distinctiveness” and finally to “internationalization.” During the 1950s “March Toward Science” campaign, policies emphasized “scientizing TCM.” By the early 21st century, rising “cultural confidence” led to the Outline for the Innovation and Development of Traditional Chinese Medicine explicitly calling for “preserving TCM’s unique advantages.” Post-2017 TCM Law implementation saw policy discourse pivot to “promoting TCM globalization,” reflecting global governance aspirations under the “Healthy China” strategy. These ideational shifts resonate with Wang Shaoguang’s theory of “policy paradigm shifts,” uncovering the deep logic of institutional reconstruction.

The driving force behind ideational change stems from transformations in societal cognitive frameworks. For example, during the 1980s “cultural fever” movement, Fei’s (26) concept of “cultural consciousness” directly influenced policies prioritizing TCM distinctiveness. This period’s Outline for Modernizing Basic TCM Theory retained core TCM tenets while integrating modern systems science methodologies (National Administration of TCM, 1991). Entering the 21st century, globalization-era “civilizational dialogue” ideals propelled policy internationalization. The 2015 Plan for International Standardization of TCM set a target of “leading 300 international TCM standards,” demonstrating institutional entrepreneurs’ strategies to gain global influence through ideational reconstruction. This ideational shift aligns with Meyer and Rowan’s (27) “institutional myth” theory—legitimacy is enhanced by promulgating universalistic ideas like “the modern value of traditional medicine.”

## Conclusion

This study systematically examines the evolutionary logic of Chinese traditional Chinese medicine education policies since 1949 by combining the theoretical framework of historical institutionalism with NVivo-based quantitative analysis. The research has found that the development of policies shows a four-stage evolution characterized by “Standardization-Characterization-Systematization-Standardization.” The core driving forces behind this evolution stem from institutional path dependence, the impact of crucial historical junctures, the game among multiple stakeholders, and the generational shift of dominant concepts. NVivo data analysis reveals that the policy keywords have gradually shifted from early terms such as “education” and “scientification” to “internationalization” and “high-quality development,” confirming the strategic transformation from building domestic institutions to participating in global governance.

The study reveals that the changes in TCM education policies essentially represent the state’s institutional reconstruction of the value of traditional culture during the process of modernization. In the early stage, the integration of the traditional apprenticeship system into the modern education system through institutionalization not only established the legitimate status of TCM but also formed an effect of path dependence, making the “integration of traditional Chinese and Western medicine” a long-term policy keynote. After the reform and opening up, economic transformation and cultural self-awareness propelled the policy to shift towards “distinctiveness cultivation.” The promulgation of the New Healthcare Reform in 2009 and the Traditional Chinese Medicine Law in 2017 marked a transition from administrative dominance to legal protection, and policy tools gradually became more diversified. It is worth noting that the proposal of the “Healthy China” strategy against the backdrop of globalization has prompted the policy discourse system to shift from “cultural protection” to “international standard export,” reflecting the efforts of institutional innovators to gain global discourse power through the reconstruction of concepts.

The theoretical value of this study lies in expanding the explanatory boundaries of historical institutionalism and demonstrating the unique logic of institutional evolution in a non-Western context. That is, under the leadership of the state, the modern transformation of traditional cultural education is achieved through the selective inheritance of traditional resources, the strategic response to external shocks, and the dynamic balance of the demands of multiple stakeholders. The research results have practical implications for the development of TCM education: it is necessary to strengthen the reform in key areas (such as the reconstruction of the clinical education system) while maintaining the continuity of the system; establish a cultural core protection mechanism during the process of internationalization; and solve the dilemma of “policy suspension” through cross-departmental collaborative innovation. Future research can further explore the potential impact of digital technology on the paradigm of TCM education and the issue of cultural adaptability during the process of institutional transplantation.

### Strengths and limitations of this study

A mixed-methods approach was adopted, combining quantitative content analysis of 197 policy texts using NVivo software with qualitative analysis under the theoretical framework of historical institutionalism. This integration enhances the scientific rigor and explanatory power of the research.

The study spans over 70 years of policy evolution (1949–present), divided into four historical stages, to systematically reveal the dynamic interplay between TCM education policies, China’s modernization process, and waves of cultural self-awareness, providing a comprehensive macro-historical narrative.

The analytical framework incorporates diverse stakeholders, including government agencies (e.g., NATCM, MOE), academic associations, and international organizations, uncovering the driving mechanisms of interest group dynamics and compromise in policymaking.

The reliance on publicly available official policy documents may overlook unpublished decision-making processes (e.g., internal meeting records or informal negotiations), leading to incomplete interpretations of policy evolution dynamics.
